# Assessing the impact of counseling HTLV-1 seropositive women on vertical transmission in the GIPH cohort study, Brazil

**DOI:** 10.1186/1742-4690-11-S1-P52

**Published:** 2014-01-07

**Authors:** Mariana A de Souza, Cláudia LF Horiguchi, Gabriela S Freitas, Rafael HC Bastos, Débora B Reiss, Marina L Martins, Maria Sueli N Lopes, Anna Bárbara FC Proietti

**Affiliations:** 1 Faculdade da Saúde e Ecologia Humana (FASEH), Vespasiano, Minas Gerais, Brazil; 2 Interdisciplinar de Pesquisa em HTLV (GIPH), Belo Horizonte, Minas Gerais, Brazil

## 

HTLV-1 is endemic in Brazil and is associated with major illnesses (HAM/TSP, ATL and uveitis). Since the infection remains asymptomatic in the majority of cases and screening is only performed in blood banks, there is an ongoing vertical transmission that remains unnoticed by the public health system. Although recommended by Brazilian researchers of the field, the prenatal screening for HTLV-1 or 2 was not yet implemented throughout the country. We performed a cross-sectional analysis to verify the impact of counseling HTLV-1 seropositive women in childbearing age participating in the GIPH cohort. The counseling included recommendations of not breastfeeding, giving infant formula and preferably having the child delivered by cesarean section. Children born from HTLV-1 seropositive women were divided in two groups: (1) born before and (2) after the participation of mothers in the GIPH cohort (“GIPH babies”). We have identified 21 children born to HTLV-1 positive mothers who received no counseling and 18 “GIPH babies”, born from mothers who received counseling. 3/21 (14.3%) and 1/18 (5.6%) of the children were found positive for HTLV-1. The numbers will be expanded with active search of the remaining children not yet tested (n=15). Although the numbers are still preliminary, they point to a trend of successful counseling and avoidance of transmission of the virus. These actions should be widespread in the country to avoid the silent transmission of the virus.

